# Successful preterm pregnancy in a rare variation of Herlyn-Werner-Wunderlich syndrome: a case report

**DOI:** 10.1186/s12884-018-2133-2

**Published:** 2018-12-17

**Authors:** Stefania Cappello, Eleonora Piccolo, Francesco Cucinelli, Luisa Casadei, Emilio Piccione, Maria Giovanna Salerno

**Affiliations:** 1 Department of Biomedicine and Prevention, Obstetrics and Gynecological Clinic, University of Rome “Tor Vergata”, via Montpellier 1, 00133 Rome, Italy; 2 Department of Woman’s and Child’s Health, Obstetrics and Gynecological Unit, San Camillo-Forlanini Hospital, Circonvallazione Gianicolense 87, 00152 Rome, Italy

**Keywords:** Herlyn-Werner-Wunderlich syndrome (HWWS), OHVIRA syndrome, Müllerian duct anomalies, Ectrodactyly, Renal agenesis, Utero didelphys, Obstructed hemivagina, Pregnancy

## Abstract

**Background:**

Herlyn–Werner-Wunderlich syndrome (HWWS) is an uncommon congenital anomaly of the female urogenital tract, characterised by uterus didelphys, obstructed hemivagina, and ipsilateral renal agenesis. We reported the difficult pregnancy course complicated by an extremely rare and unique case of this syndrome associated with ectrodactyly, a clinical combination never described in literature.

**Case presentation:**

A 28- year-old nulliparous woman previously diagnosed for HWWS associated with ectrodactyly of the right foot and with a history of abdominal left hemi-hysterectomy, ipsilateral salpingectomy, vaginal reconstruction when she was an adolescent. She suffered from threats of abortion in the first trimester, recurrent urinary tract infections during all pregnancy. At 33 weeks + 5 days of gestational age, she was hospitalized for premature rupture of the membranes and uterine contractions and a caesarean section was performed because of breech presentation. Postpartum period was complicated by a pelvic abscess resolved with parental antibiotic therapies.

**Conclusions:**

Our literature review shows an unusual aspect in our case: HWWS is not classically associated with skeletal anomalies. Moreover, the most frequent urogenital side affected is the right, not left side as in this woman. Preterm spontaneous rupture of membranes and fetal abnormal presentation represent frequent complications and probably post-caesarean infections are related to pregnancies in the context of this syndrome.

## Background

Herlyn –Werner-Wunderlich syndrome (HWWS) is a rare congenital anomaly of the female genital tract, characterised by the triad of uterus didelphys, blind hemivagina, and ipsilateral renal agenesis [1, 2]. It is even called OHVIRA syndrome (obstructed hemivagina, ipsilateral renal anomaly) and it is a result of the arrest of the midline fusion of the Müllerian ducts. This condition was first described in 70’s and the incidence is low and probably underestimated [3]. Usually the diagnosis comes during adolescence due to hematocolpos, the frequent urinary tract infections or the presence of a pelvic mass [4, 5]. Although all the Müllerian anomalies are related to infertility, frequent miscarriage, obstetric complications including abnormal fetal presentation, intrauterine growth restriction, and increased rate of caesarean section as well as abruptio placentae, premature rupture of membranes, retained placenta, postpartum haemorrhage, and increased fetal mortality [6, 7]. We presented a case of an unusual variant of HWWS associated with ectrodactyly [8] of the right foot, a combination never described [9], and the difficult course of her first preterm pregnancy with a particular postpartum complication. We are not sure if the ectrodactyly is an incidental finding or there is a causal effect with the disease, because it was not described by literature before.

## Case Presentation

**Fig. 1:**
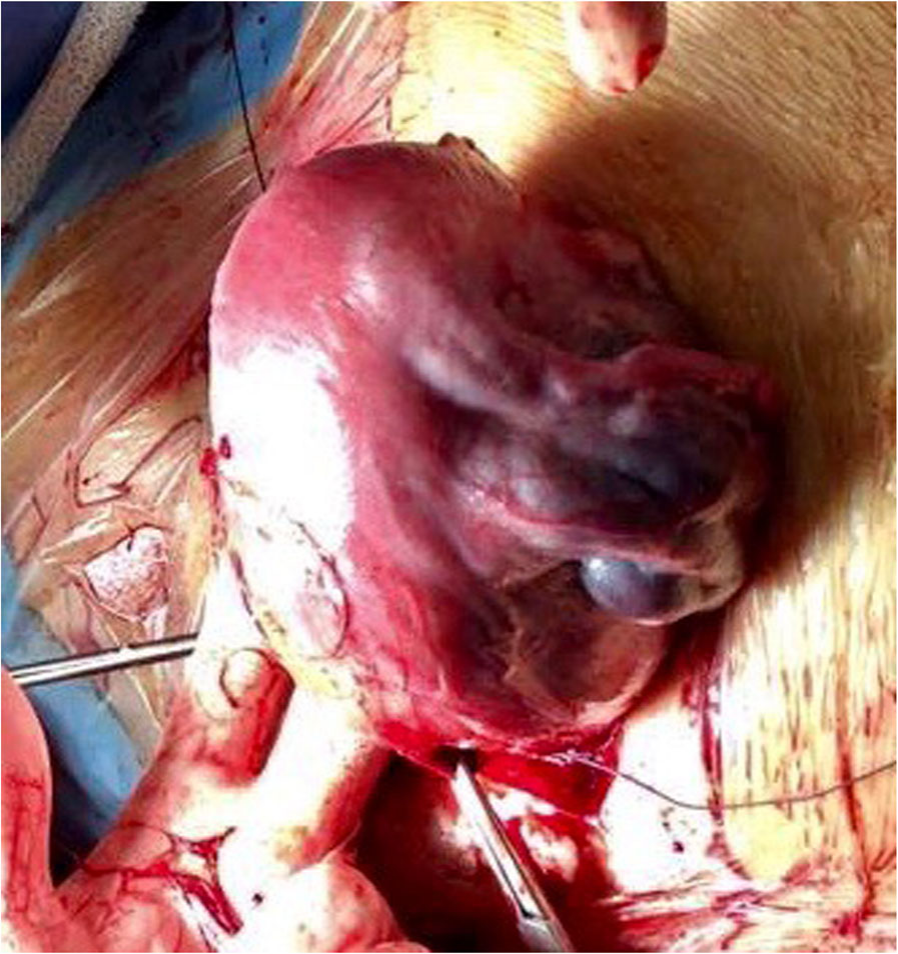
Uterus at the time of caesarean delivery

**Fig. 2:**
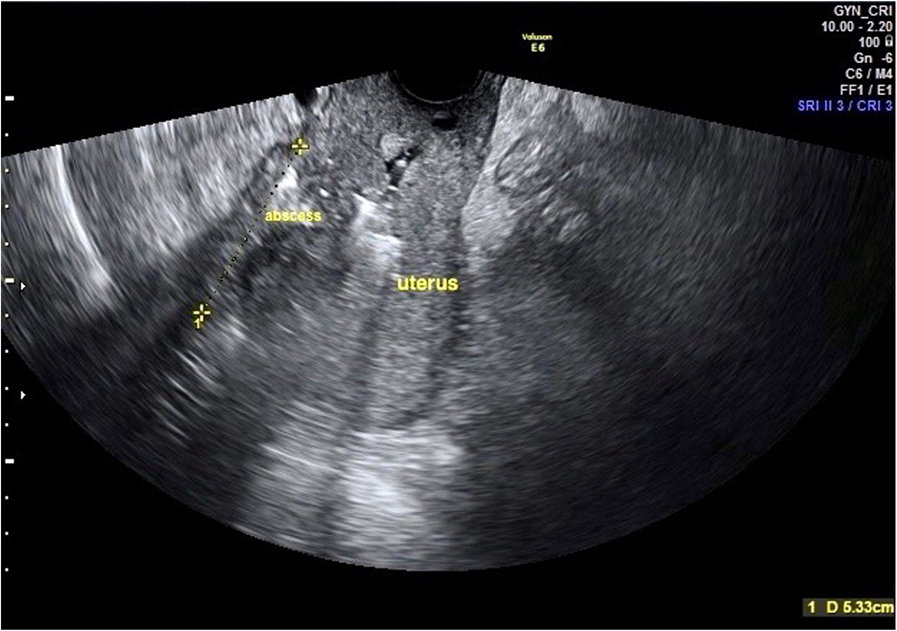
Pelvic Abscess at the ultrasound imaging. Abscess neighboring to the lower anterior wall of the uterus with dimensions of 53 x 47 mm

A 28 year-old nulliparous woman, previously diagnosed for HWWS, was referred to San Camillo-Forlanini Hospital during her third spontaneous pregnancy. The clinical history of our patient began when at her birth, the ectrodactyly of the right foot (absence of the 2 medial rays), immediately became apparent. The karyotype analysis was normal 46 XX. At age 1, she underwent a surgical correction of this anomaly with consequent partial improvement of a functional deficiency. An upper abdominal ultrasound, performed after a history of recurrent urinary tract infections and pyelonephritis, revealed the absence of the left kidney and the right megaureter. At 12 years, after 2 months from menarche, due to severe acute pelvic pain, a pelvic ultrasound and a magnetic resonance imaging (MRI) were performed. MRI showed a left blind hemivagina with hematocolpos, uterus didelphys with hematometra in the left hemiuterus and ipsilateral hematosalpinx. These imaging findings were later confirmed by the diagnostic laparoscopy which showed normal right uterus, right fallopian tube and both regular ovaries. Consequently, she underwent a surgical reconstruction of the vagina consisting in the drainage of hematocolpos and the removal of the vaginal septum, whereas an abdominal left hemi- hysterectomy and ipsilateral salpingectomy were performed through a Pfannenstiel incision. Her obstetric history was significant for two spontaneous abortion at the age of 26, occurred at 7th and 12th weeks respectively. She had no problem of fertility in anamnesis. The woman came to our observation for the first time at 15 weeks of pregnancy for abortion threats resolved with vaginal progesterone. Singleton fetus was anatomically in the norm. The patient had a moderate protenuria of 1400 mg in 24 hours, so she started a proper diet and a monitor of urine proteins. Close and regular surveillance (clinical, laboratory, and ultrasound) was initiated. The obstetric ultrasound controls revealed adequate growth of a fetus without major malformations, and normal Doppler indices of the fetal, feto-maternal and utero-placental vessels. During the three trimesters, frequent urinary infections occurred that were appropriately treated after urine culture and antibiogram test. At 33 weeks + 5 days of gestational age she was admitted to our hospital for premature spontaneous rupture of membranes (pPROM): she reported light amniotic fluid leak one hour before our observation. The admission assessment detected a reduced amniotic fluid index, a regular fetal growth and posterior placenta in the norm; the umbilical artery Doppler values were in the range, the fetal cardiac monitoring was regular and uterine contractions were present. The vaginal examination revealed a soft cervix, 80% effaced, dilated about 2 cm, and the fetus was in breech at station -3. The ultrasound cervical length was 24 mm. A single course of antenatal corticosteroid therapy for fetal lungs maturity induction and tocolytic drugs were administered. On the second day of hospitalization, an emergency caesarean section was performed because the cervix was modified (dilatation about 4 cm), uterine contractility increased while persisting breech presentation (Fig. 1). A female infant was born weighing 2278 gr, with Apgar scores 8/9/9 at 1, 5, and 10 minutes respectively; the umbilical artery ph was 7.35. The placenta weighed 380 grams. The mother and the newborn, made an uncomplicated post-surgical/postnatal course and were discharged on day 3 and 15 respectively. Seventeen days after caesarean section, the woman came back again to our institution for a complaint of asthenia and fever ( > 38°C), resistant to paracetamol for five days. On physical examination, she had abdominal tenderness in the lower quadrants and physiological vaginal lochia. Blood exams showed increased leukocyte and inflammatory markers: white blood cells (WBC) were 14,2 x 10^3^/ml (range 4,0-10,0 x 10^3^/ml); C-reactive protein (CRP) was 19.98 mg/l (range 0,01-1 mg/dl) and procalcitonin was 0.22 ng/ml (negative: <0,05 ng/ml). The pelvic ultrasound and the computerized tomography (CT) demonstrated a pelvic abscess neighboring to the lower anterior wall of the uterus with dimensions of 53 x 47 mm (Fig. 2). The treatment started immediately and consisted in intravenous antibiotic therapy with Meropenem 500 mg three times a day and low- molecular weight heparin (LMWH), Enoxaparin 4000 UI subcutaneous daily. Six days after the hospital admission with the right therapy, the inflammatory indices reduced: WBC were 9,8 x 10^3^/ml, PCR was 4.37 mg/l and procalcitonin was 0.12 ng/ml. After discharge, we started a follow-up to assess the clinical conditions of our patient: she was asymptomatic, her blood exams were within normal ranges and the pelvic abscess was significantly decreased. Histopathologic examination of the placenta and umbilical cord obtained after about 40 days, not identified signs of chorioamnionitis and/or funisitis.

## Discussion

Müllerian duct anomalies affect 2-3% of women [10]. The combination of uterus didelphys -class III of American Fertility Society –AFS- classification [11] and obstructed hemivagina was described the first time in 1922 [12], then in 70’s Herlyn, Werner and Wunderlich reported other similar cases associated with renal anomalies [1, 2]. In 1983 this condition was identified as a syndrome characterised by the triad of uterus didelphys, blind hemivagina and ipsilateral renal agenesis [13]. Since 2007 this syndrome was named with the acronym of OHVIRA syndrome (obstructed hemivagina, ipsilateral renal anomaly), including two of the three of defects of the HWWS, which consists of different uterus anomalies (uterus didelphys or uterus septum) and renal anomalies as well as renal agenesis or polycystic kidney [14–16]. The occurrence of the HWWS seems to be 0.1-3.8%, and it is probably underestimated [3]. HWWS was included in the class U3B uterine anomaly, class C2 cervix anomaly, and class V3 vaginal anomaly according to the classification of ESHRE/ESGE [17]. The exact etiology of HWWS is still unclear, but it may be related to an abnormal development of the para- and mesonephric ducts. Didelphys uterus results from the failure of the fusion and differentiation of the Müllerian ducts during the eight week, which should give rise to cervix and uterus [18]. The Wolffian ducts give rise to ureters and kidneys, so when one of these is absent, the ureter and the kidney cannot fuse and the ipsilateral Müllerian duct is lateralized moving away from the urogenital sinus, causing the formation of a blind sac that will correspond to the blind or obstructed hemivagina. The distal portion of the vagina originating from the urogenital sinus is not affected and develops normally. Patients affected by HWWS have no specific symptoms until puberty [5], then they typically present acute pelvic pain, dysmenorrhea, presence of pelvic mass, recurrent urinary tract infections and urinary retention. The diagnosis usually comes in the adolescence, a few years after the menarche, rarely at the birth or during pregnancy [19, 20]. In fact there is only a case described in literature by Wu et al. of a neonate diagnosed for HWWS presenting as a mass prolapsing per vaginum [21]. Clinical suspicion and early diagnosis are imperative to making a timely treatment to prevent complications such as infertility, endometriosis, pelvic adhesions, pyosalpinx, hemato or pyocolpos, and other obstetrics problems [22]. MRI was considered the gold standard for the diagnosis, but the 3d ultrasound plays always a major role for the identification of uterine anomalies [23]. Diagnostic laparoscopy in HWWS should performed only when the diagnosis by imaging is not clear or when MRI is not available [5, 24]. Twenty-five percent of women affected by Müllerian ducts anomalies presents obstetric complications such as recurrent miscarriage, abnormal fetal presentation, postpartum hemorrhage, retained placenta, fetal mortality, fetal growth restriction, premature rupture of membranes [7]. Obstetric outcome of HWWS women was studied after different surgical treatments. These may consist of conservative treatments like the desobstruction of hemivagina, the therapeutic drainage of hematocolpos, the vaginal septotomy and marsupialization; or less conservative interventions like laparoscopic hemi-hysterectomy associated or not with ipsilateral salpingectomy [25–28]. The best treatment of HWWS is controversial but most of the authors conclude that an explorative laparoscopy with vaginal septotomy and drainage of hematocolpos is enough to restore the functionality of both part of the uterus, avoiding hemi-hysterectomy [29, 30]. However, HWWS has good obstetric prognosis: 87% of pregnancy rate [27], approximately 62% positive obstetric outcomes without complications during delivery [5, 7]. Haddad reported the reproductive performance of 42 patients with obstructed hemivagina, 9 of whom had 20 pregnancies with 69% of live births [31]. Heinonen reported the reproductive performance and obstetric complications of 49 patients affected by didelphys uterus finding that the incidence of primary infertility was not significantly increased in these women. The rate of spontaneous miscarriage was 21%, not significantly different from the general population (15-20%). Preterm birth took place in 24% of all parts. This rate is higher than that of the general population (9-10%). Instead the caesarean sections rate is 84% and it reflects the high incidence of the breech presentation (51%) [29, 32]. In the presentation of this case we drew attention to its rarity because it involves the left part of the body, while usually is the right side affected [28, 33, 34] and the association with a malformation of the skeleton of the lower limbs, ectrodactyly of the right foot, was not ever been reported in literature; we found only a case report which described the combination of HWWS and lumbar scoliosis [35] and we are not sure if the skeletal involvement could be only an incidental finding. However, not all authors agree that it is a rarity the involvement of the left side: Yavuz et al. presented a case series of 13 women affected by HWWS and 6 of them had anomalies on the left side of the uro-genital tract [36]. According to the classification proposed by Zhun et al, this case belonged to class 1.1 of HWWS with a complete vaginal obstruction for a blind hemivagina [37] and she had no fertility problems following surgery.

In our experience, we focused on the course and management of the pregnancy complicated of this disorder. The pregnancy we described was at high-risk and the surgical treatment performed during adolescence was the least recommended by literature, however the obstetric outcome was positive thanks to a straight maternal-fetal follow-up. Our case was characterized by two complications of the pregnancy: preterm labour with pPROM and breech presentation, which are frequent in HWWS in literature. Additionally, pPROM was related to uterine anomaly and probably promoted by the frequent infections of urinary tract [38] that may be present in this syndrome, [39], mostly in the pregnant female. The infection occurred post the cesarean section may be caused by the pPROM, the elongate surgery time, the numerous adhesions of the previous surgery, which are known risk factors in post-operative infections [40].

## Conclusions

HWWS is an unusual congenital anomaly with clinical significance and different options for surgical management. An early correct diagnosis and treatment are the goal to relieve symptoms and prevent complications to preserve sexual and reproductive abilities. Despite this, the pregnancies of these women are at an increased risk for unfavorable obstetric outcomes and can be characterized more frequently by complications that must be managed promptly by an accurate and regular maternal and fetal follow-up.

## Abbreviations

AFS: American Fertility Society
CRP: C-reactive protein
CT: Computed Computed tomographic
ESGE: European Society of Gynecological Endoscopy
ESHRE: European Society of Human Reproduction and Embryology
HWWS: Herlyn-Werner-Wunderlich syndrome
LMWH: Low Low molecular weight heparin
MRI: Magnetic Magnetic resonance imaging
OVHIRA: Obstructed Obstructed hemivagina ipsilateral renal anomaly
pPROM: Premature Premature rupture of membranes
WBC: White White blood cells
